# An object oriented implementation of the Yeadon human inertia model

**DOI:** 10.12688/f1000research.5292.2

**Published:** 2015-04-07

**Authors:** Christopher Dembia, Jason K. Moore, Mont Hubbard

**Affiliations:** 1 Mechanical Engineering, Stanford University, Stanford, CA, 94305, USA; 2 Mechanical Engineering, Cleveland State University, Cleveland, OH, 44115, USA; 3 Mechanical and Aerospace Engineering, University of California, Davis, CA, 95616, USA

**Keywords:** segmental inertia, open source software, human movement, aerial movement

## Abstract

We present an open source software implementation of a popular mathematical method developed by M.R. Yeadon for calculating the body and segment inertia parameters of a human body. The software is written in a high level open source language and provides three interfaces for manipulating the data and the model: a Python API, a command-line user interface, and a graphical user interface. Thus the software can fit into various data processing pipelines and requires only simple geometrical measures as input.

## Introduction

For dynamic analyses, it is typical to treat the human body as a collection of linked rigid bodies. For accurate simulation and analysis, the inertial properties (mass, center of mass location, and moments of inertia) of each of the body segments must be estimated. Human inertial properties have been measured and estimated in a number of ways. Bjornstrup ^1^ gives a detailed overview of mostly invasive methods up to 1995. Many other methods exist, including cadaver measurements ^2– 4^, photogrammetry ^5^, ray scanning techniques ^6, 7^, water displacement ^8^, rotating platforms ^9^, and geometrical estimation of the body segments ^10^.

Yeadon’s mathematical method ^10^ is attractive because it requires only a set of simple geometric measurements from a human and provides reasonably accurate estimates of an individual’s body segment parameters using simple, straightforward computations. Yeadon himself developed a Fortran program called ISEG in his doctoral work ^11^, to efficiently compute the inertial parameters. The original source code is available in his dissertation but is not adaptable for inclusion in modern software packages, is not copyrighted under a liberal reuse license (i.e., Creative Commons Attribution-NonCommercial-NoDerivs 2.5), and is not very user friendly.

Because we often make use of Yeadon’s model in our research, we developed a modern object oriented program under a permissive license that allows for incorporation into other software packages and includes a graphical user interface for ease of use.

## Yeadon’s model

In 1990 Yeadon published a four-paper series based on his dissertation work concerning simulating human aerial movement, particularly twisting somersaults. His first paper ^10^ describes a method for obtaining joint angles from film data, and accordingly develops a scheme to define the orientation of the whole body and the relative orientation of its parts. The second paper ^12^ describes in detail the geometry of the human model. The description of the model configuration is contained in the third paper ^13^, which also details the analytical calculation of the angular momentum. The last paper in the series ^14^ compares a film recording of the trajectory of an aerial flight to a computer simulation using the model developed in the first three papers.

Yeadon provides a lucid explanation of the human inertia model in the series described above. In this section, we merely seek to summarize his work. Note that we are not concerned with the angular momentum of the model; rather we are solely interested in the model’s inertial properties.

**Figure 1.**
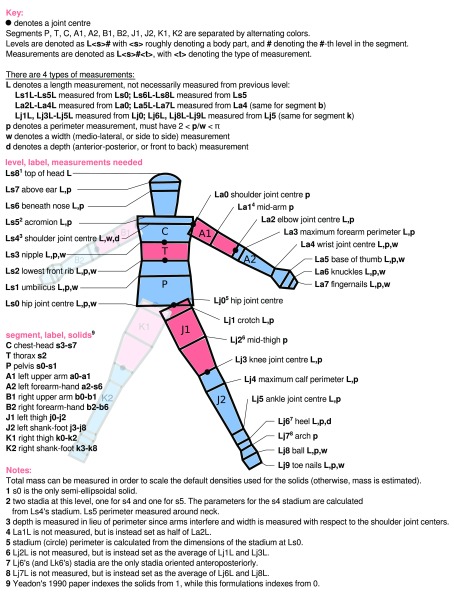
Measurements required for the human inertia model. The human is defined in terms of segments, levels, and solids. Segments are distinguished by alternating colors, and are denoted as P, A1, etc. Levels are denoted as “L<s>#”, where <s> denotes a segment of the body (e.g. j for the left leg) and # denotes the index of the level in that segment. The solid that is proximal to level L<s># is denoted as “<s>#”. The letter “s” is used for levels and solids in segments P, T, and C, as is done in Yeadon’s original work ^12^. The model is personalized via 95 measurements of 4 types: lengths L along the longitudinal axis of the segments, perimeters p about the segments, mediolateral widths w, and anteroposterior depths d. Black dots denote joint centers ^10^.

The model is defined in terms of *segments*, *levels*, and *solids*. These three elements of the model are all shown in Figure 1.

**Segments**   The human is assumed to be composed of eleven rigid segments. Each of the four limbs has two segments, and the remainder (head and torso) consist of three more. Each of these segments is a rigid body, and has at least one rotational degree of freedom with respect to the adjacent segment to which it is attached. The segments are labeled **C**, **A1**, etc.

**Figure 2.**
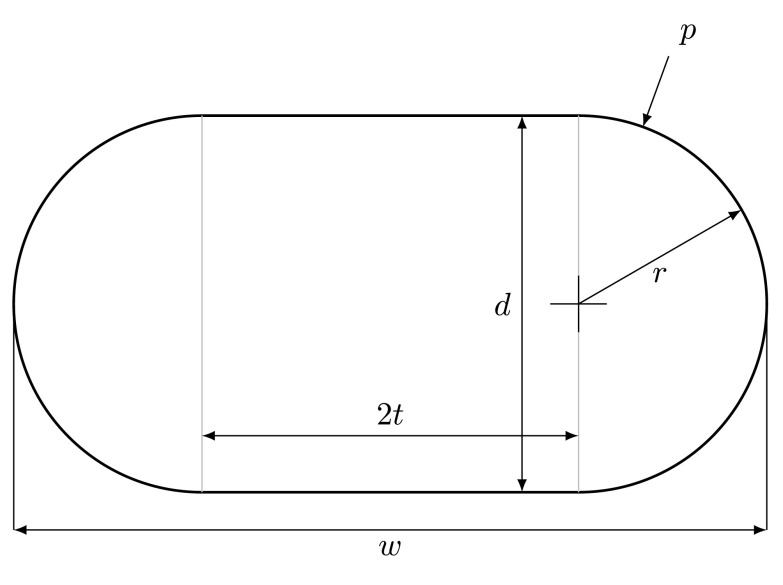
General stadium cross section shape. The levels that define the segments are all in the shape of a stadium, with one exception. A stadium is defined by any two of its attributes: perimeter *p*, radius *r*, thickness *t*, width *w*, and depth *d*.

**Levels**   Each segment is defined by a series of parallel transverse cross sections, referred to as levels, both in Yeadon’s work and in ours. The model contains a total of 45 levels, labeled **Ls0**, **La0**, etc. Each level has the shape of a *stadium* (see Figure 2). A stadium can be defined by any two of the following five attributes: its perimeter *p*, radius *r*, thickness *t*, width *w*, and depth *d*. However, there are a few situations in which the stadium degenerates into a circle. The choice of which two attributes are used to define a given stadium depends on its location in the body. For example, it is difficult to measure a perimeter around the shoulders ( **Ls4**), so a depth is used instead.

**Solids**   The inertial properties of each segment are computed by viewing each segment as a solid lofted through all the levels in the segment. This defines *N −* 1 solids in a segment with *N* levels. The solids are labeled **s0**, **a0**, etc. All solids in a segment share the same longitudinal axis. The shape of a given solid is defined by its two bounding stadia and the longitudinal distance between them (the solid’s height). These are termed *stadium solids*. The only exception is **s7**, the solid above the ear, which is a semi-ellipsoid. The model contains a total of 40 solids. Note that in this formulation we begin numbering the solids from 0, while Yeadon numbers the solids from 1. This is simply to match Python’s 0-based indexing.

A key feature of this inertia model is that it can be personalized to a given individual (it is subject specific). The model is personalized via 95 anthropometric measurements. These serve to define each stadium, and to specify the distances between the stadia (the heights of the stadium solids).

**Figure 3.**
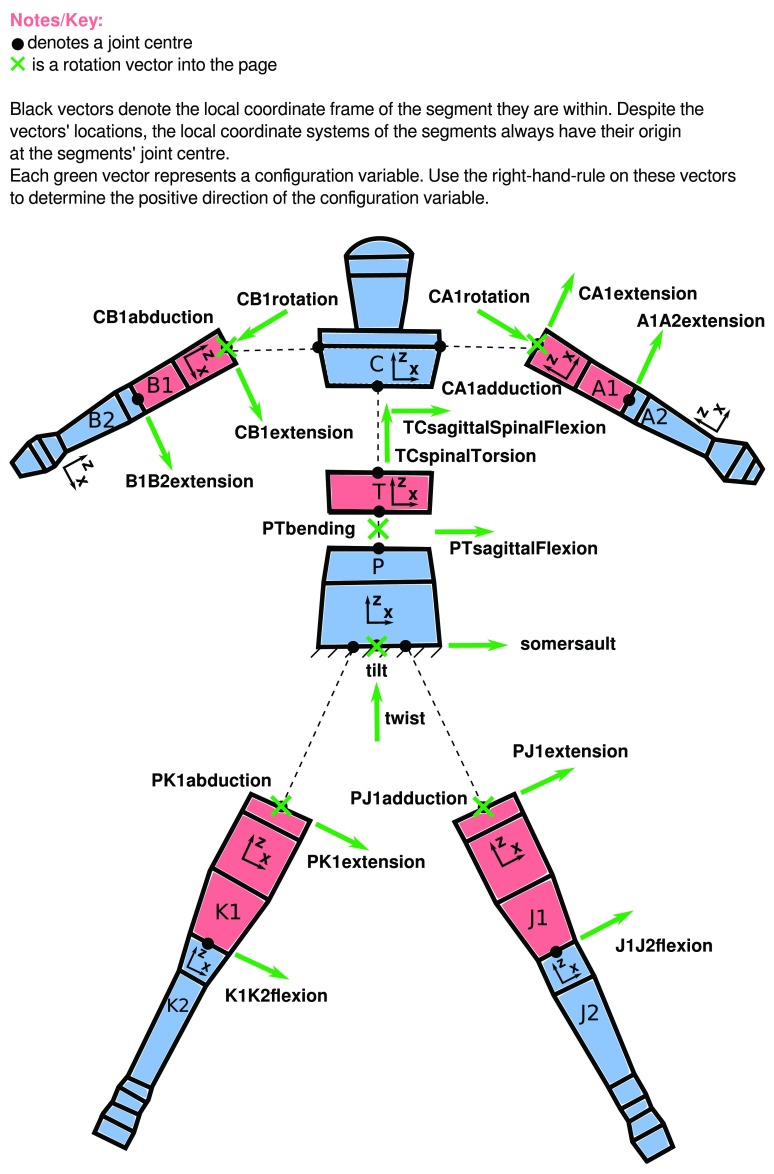
Configuration variables for the human inertia model. The configuration of the human is defined using 21 joint angles, represented by green vectors. Green crosses and circles represent vectors into and out of the page, respectively. The direction of rotation for a joint angle is given by the right-hand rule about its vector. Labels beside the vectors are the names of the configuration variables in the code. The black dot on each segment denote its joint center. The origin of the fixed coordinate frame is at the bottom center of the pelvis segment. The local coordinate frame of each segment is specified by the pair of perpendicular black vectors in or next to the segments. Despite the locations of these black vectors, the origin of a segment’s local coordinate system is always at its joint center ^13^.

In addition, much of the model’s utility comes from the ability to specify the configuration of the human in which the inertial properties are desired. The configuration is specified using 21 joint angles between the various segments; these are described in Figure 3.

Thus, Yeadon’s model is defined via segments, levels, solids, and configuration angles. One can personalize the model to an individual using measurements, and obtain inertial parameters for this individual in any desired configuration. The only additional data needed are the densities of the solids. We use Dempster’s segmental densities ^2^ by default, as does Yeadon. But the user has the option to reassign these to alternative values if desired. The model is then completed with analytical expressions for a stadium solid’s and semiellipsoid’s center of mass location and moments of inertia, using the parallel axis theorem to combine the inertial properties of multiple stadium solids. Yeadon provides these explicit formulae in ^12^. In the next section, we describe in more detail the measurements required for the model and the way the configuration is defined.

## Implementation of Yeadon’s model

The previous section makes no departures from Yeadon’s work. However, we will now make some changes that are important for computational implementation and that serve to generalize his work. These are summarized at the end of this section.

The experimentalist provides all of the measurements, and optionally the configuration angles, in two human readable YAML formatted text files. The details of these inputs follow.

### Measurements

Measurements are of four types: lengths ( **L**), perimeters ( **p**), widths ( **w**), and depths ( **d**) and are always tied to a level. For example, the symbol **La1p** denotes the perimeter at the **La1** level.

Although we require the heights of the individual stadium solids, the experimentalist does not measure these independently. Instead, measurements are made of the longitudinal distance across multiple stadium solids. For example, the **Ls2L** measurement is not the distance between the **Ls1** and **Ls2** levels. Instead, **Ls2L** is measured as a distance from **Ls0**. To learn the levels from which one measures the various lengths, see Table 1.

**Table 1.** Length measurements.

length for these levels	are measured from this level
Ls1 - Ls5	Ls0
Ls6 - Ls8	Ls5
La2 - La4	La0
La5 - La7	La4
Lb2 - Lb4	Lb0
Lb5 - Lb7	Lb4
Lj1, Lj3 - Lj5	Lj0
Lj8 - Lj9	Lj5
Lk1, Lk3 - Lk5	Lk0
Lk8 - Lk9	Lk5

The length measurements are not simply the heights of the stadium solids. They are defined relative to a certain preceding level in their segment.

There are a few exceptions to the general measurement scheme we have described thus far. While most of the lengths are measured directly, some are determined by other lengths. For example, we set **La1L** to be half of **La2L**, and so the experimentalist does not measure **La1L** directly. This means, however, that **La1p** must be measured halfway down segment **A1**. This scenario arises in each limb.

All stadia are oriented mediolaterally except the heels (levels **Lj6** and **Lk6**) which are oriented anteroposteriorily. Note from Figure 1 that one measures a depth at these levels instead of a width. This depth is in fact the width of a stadium that is rotated through 90 degrees. Other necessary information about exceptions in the measurement scheme is contained in the notes of Figure 1.

Since the densities for the model are provided, one can readily estimate the human’s total mass. However, if the experimentalist measures the mass of the subject, that mass can be used to proportionally scale all densities in the model so that the model’s total mass matches the subject’s measured mass. Be aware that scaling the densities by the total mass is not a guarantee that errors in all the inertial estimates are reduced.

### Configuration

In this section we describe, relying heavily on Figure 3, how the configuration of the model is implemented. The joint center of each segment is located with a black dot: it is always located at the center of the base stadium of the segment. The black arrows on each segment indicate its local coordinate frame, whose origin is always at the joint center of the segment. For each segment, the local *z*-axis is the longitudinal axis of the segment. Each of the green arrows represents a degree of freedom, and indicates the direction and sign of the corresponding joint angle via the right-hand rule. Configuration variables are labeled with the names of the two segments at the joint, and a physiological description of the joint angle. Thus, K1K2flexion is the right knee flexion angle, and PK1abduction is the right hip abduction angle (positive for abduction, negative for adduction), etc. The exceptions to this naming convention are somersault, tilt, and twist, which specify the orientation of the **P** segment with respect to the fixed coordinate frame.

Most joints enable more than one degree of freedom, but only four have all three rotational degrees of freedom. Since rotations are not commutative, we must specify the order of rotations at multi-degree of freedom joints. Each child segment is rotated relative to its parent segment using Euler X-Y-Z angles in a body fixed fashion. Any joints with fewer than three angles follow this same order, e.g. X-Y.

The default configuration is that in which all configuration variables have a value of zero. In the default configuration, the local coordinate basis vectors of all segments align with the global fixed coordinate basis vectors. This means that for each segment, in the default configuration, the local *x*-axis lies in a coronal plane and the local *y*-axis is directed posteriorly. Furthermore, it is assumed that in this configuration, the palms of the hands face anteriorly. We now provide the locations of joint centers in our implementation of Yeadon’s model; this information is not in his original papers. The location of the joint centers of segments **A1** and **B1** are at the most distal points of level **Ls4** on the respective sides of the body. Joint center locations for segments **J1** and **K1**, respectively denoted as **p _J_** and **p _K_**, lie within level **Ls0** and can be expressed in the local coordinate frame of the pelvis **P**:

$$p_{J}=\frac{1}{2}\left(t_{\text{Ls0}}+r_{\text{Ls0}}\right)\hat{i}\;\;\;\;\;\;(1)$$

$$p_{K}=–\frac{1}{2}\left(t_{\text{Ls0}}+r_{\text{Ls0}}\right)\hat{i},\;\;\;\;\;\;(2)$$

where $\hat{i}$ is a unit vector along the *x*-axis of the pelvis, *t*_Ls0_ is the thickness of the stadium at level **Ls0** and *r*_Ls0_ is its radius. This choice is informed by calculations present in the ISEG code published in Yeadon's thesis ^11^; in fact, Yeadon defines the origin of the model (and of level **Ls0**) as the midpoint of the two hip joint centers. Joint center locations in all other segments are at the center of the last stadium in the preceding segment.

The model does not contain joints at the wrist or ankle, which is consistent with Yeadon’s model ^13^. Since these joints have a small effect on the inertial properties of the whole body, the exclusion of these joints should be fine for many use cases. However, the modular structure of the software allows one to easily modify the software to create these joints, if necessary.

### Departures from Yeadon’s work

There are a few ways in which our implementation of the human inertia model differs from that presented in Yeadon's papers ^10, 12– 14^. Some of these differences arise from the fact that his work was tailored for aerial movement, more specifically for twisting somersaults. We expect, however, that our implementation of the model can be used in a more general set of investigations.

**Symmetry of limbs**   Yeadon averages the measurements for the left and right limbs so that the model is symmetric. We provide the user with the option of imposing this symmetry, but the averaging is not performed by default.

**Acromion stadia**   One can see that there are actually two different cross sections at the acromion level **Ls5**: we use the wider one for solid **s4** in the chest and the thinner one (actually, a circle) for solid **s5** in the head. The perimeter measurement at **Ls5** is used for the bottom of **s5**. In our implementation, the stadium at the top of **s4** is determined internally by the **Ls4** stadium by:

$$r_{\text{Ls5}}=0.57r_{\text{Ls4}}\;\;\;\;\;(3)$$

$$t_{\text{Ls5}}=\frac{1}{2}w_{\text{Ls4}}-\text{​}r_{\text{Ls5}}\;\;\;\;\;(4)$$

where *r*_Ls5_ and *t*_Ls5_ are the radius and thickness for the top stadium of **s4**, respectively, and *r*_Ls4_ and *w*_Ls4_ are the radius and width of the stadium at level **Ls4**, respectively. This issue is not addressed in Yeadon's papers ^10, 12– 14^, and our implementation disagrees with the ISEG code found in Yeadon's thesis ^11^ (see page 358 line 251). The justification for our choice is to agree with a more recent version of Yeadon’s code, provided to us in a personal communication.

**Hip joint center stadia in the thigh**   The experimentalist makes no measurements at the **Lj0** or **Lk0** stadia, though these stadia must be defined in order for solids **j0** and **k0** to be defined. In our implementation, these stadia are circles with the same radius:

$$r_{\text{Lj0}}=r_{\text{Lk0}}=\frac{1}{2}\sqrt{r_{\text{Ls0}}w_{\text{Ls0}}}\;\;\;\;\;(5)$$

where *r*_Ls0_ and *w*_Ls0_ are the radius and width of the **Ls0** stadium, respectively. As with the acromion stadia mentioned above, the justification is that this has been implemented in the more recent version of the code shared with us.

**Relationships between configuration variables**   Yeadon enforced relationships between certain configuration variables, such as symmetric movement of the legs with respect to the pelvis. We neither assume nor impose any relationships between the 21 joint angle configuration variables; all are independent. As a result our model allows the body to assume any geometrically feasible configuration with physiological bounds.

**Inconsistent measurements**   The ratio of a stadium’s perimeter to its width must be greater than 2 and less than *π*. If the measurements do not satisfy these constraints, then the stadium is assumed to be a circle. This scenario is not discussed by Yeadon.

**Degenerate stadia**   In the case where a stadium has zero thickness (a circle), the stadium is degenerate and some equations have a zero in the denominator; however, the resulting shape is still valid. In this scenario, Yeadon still employs the formulae for stadium solids but sets the thickness to be very small ^12^. Instead, we manipulate the equations so that the approximation is not necessary. In the case that only one of the stadia for a solid has zero thickness, the divide-by-zero issue is removed by computing the mass properties for the stadium solid in which the two stadia are swapped. However, this manipulation does not work if both stadia have zero thickness; for this case, we compute the inertial properties of a truncated cone; see code for details.

**Joint center of chest-head segment**   We locate the joint center between the torso **T** and the chest-head **C** at the center of level **Ls3**. This is in accordance with Figure 1 of Yeadon's 3rd 1990 paper ^13^. However, this is a departure from the code in Yeadon's thesis ^11^, in which the joint center of the chest-head is placed at the midpoint of the shoulder joint centers.

## Software design

We implemented the inertia model in the Python programming language ^15^ as a package named yeadon (yeadon only supports Python 2.7). Python was chosen due to its ease of use, wide adoption, its stable infrastructure for distributing open source software, and its strong scientific community ( SciPy).

The input to yeadon consists of (1) geometric measurements of a subject, and (2) joint configuration angles. Using these two inputs, the inertial properties of the subject in this configuration can be calculated.

**Figure 4.**
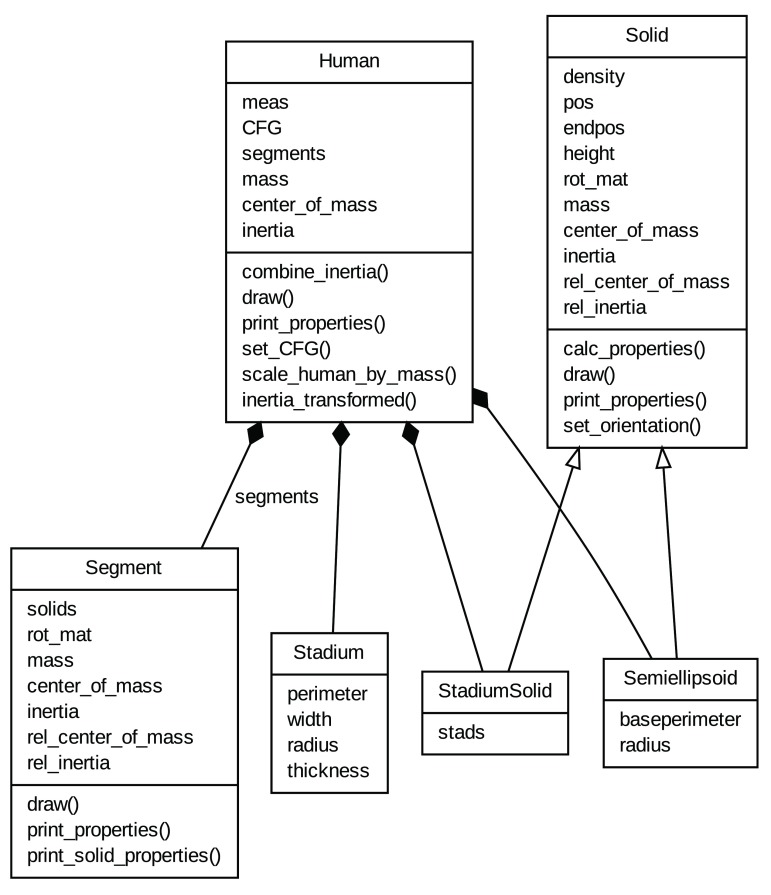
UML diagram of Yeadon package. The Human class constructs all Segment’s, Solid’s, and Stadium’s, and assembles them appropriately. The classes StadiumSolid and Semiellipsoid inherit from Solid. The user interacts with the software through the attributes or methods of the Human class, or via the ui or gui modules. This diagram does not reveal the entire public interface of the classes shown. The mass properties center_of_mass and inertia (tensor) are expressed in the global frame; quantities prefixed with rel are expressed in the Segment or Solid frame. The attribute CFG contains the configuration of the Human. See yeadon’s documentation for more information.

**Figure 5.**
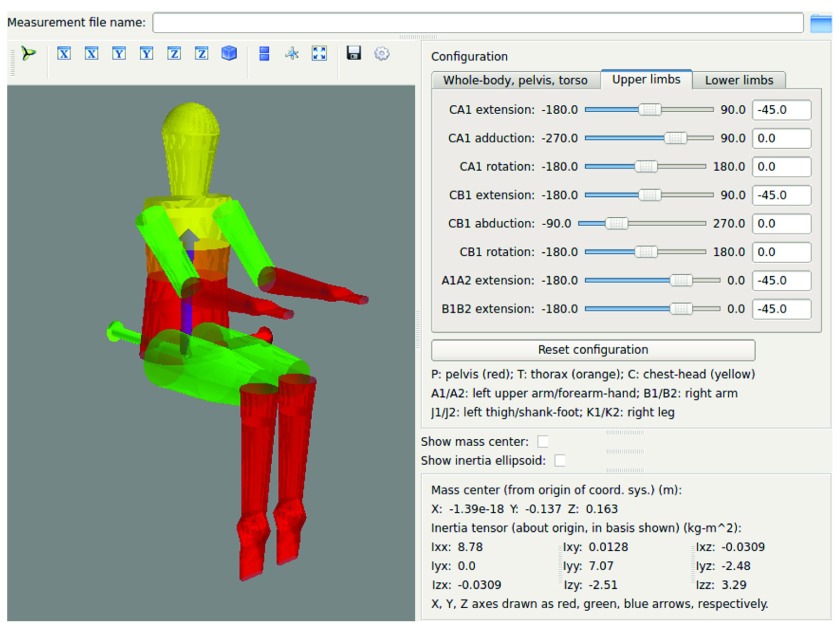
Screenshot of the GUI application. A screenshot of the Mayavi GUI application (QT backend) running on Ubuntu 14.04 is shown. The user can supply the path to measurement YAML files to obtain inertial properties for a specific subject. The graphics window on the left shows a 3D view of the model. The viewing angle, camera perspective, and other details can be manipulated with a mouse or from the toolbar above the window. Tabs on the right provide sliders and text inputs for all 21 configuration variables and allow the user to interactively set the configuration. The configuration limits attempt to prevent unphysiological configurations and do not necessarily reflect the range of motion of the subject. The “Reset Configuration” button sets all the configuration variables to zero. The mass center and inertia ellipsoid for the entire body can be toggled with the checkboxes. Finally, the whole-body mass, center of mass coordinates, and inertia tensor are displayed in the bottom right and are updated interactively as the configuration is altered.

The yeadon package contains 5 modules: human, segment, solid, ui, and gui. The human module contains the public interface of the package, and the segment and solid modules are used internally to construct objects available in the human module. The user interacts either directly with the human module, or via the ui or gui modules, both of which are clients to the human module. The human module contains only the Human class, the segment module contains only the Segment class, and the solid module contains the Stadium, Solid, StadiumSolid, and Semiellipsoid classes. The package relies heavily on composition. The Human constructor constructs all Stadium’s, Solid’s, and Segment’s, and ties together these objects appropriately. A visual description of the class hierarchy is shown in the UML diagram in Figure 4. The GUI is built using Mayavi ^16^ which is both a high level Python interface to the Visualization Tool Kit ^17^ and a lightweight application framework. We utilized Mayavi’s ability to rapidly create cross platform graphical interfaces to expose the underlying yeadon classes through interactive widgets. The graphical user interface shown in Figure 5 allows the user to load measurement data files, adjust configuration variables interactively, view the human body’s mass center location, visualize its inertia ellipsoid, and view the resulting whole-body inertial properties.

The primary outputs of the software include:

- Whole-body inertial properties of the model in any given configuration with respect to any point and reference frame.
- Segment-fixed inertial properties for any single solid or any combination of solids.
- Visual depiction of the model in a given configuration. These can be exported to bitmap files.

The software provides inertial properties (mass, center of mass location, and moments of inertia) for the whole human, for an individual segment, for an individual solid, or for any combination of segments and solids. In the first case of the whole human, center of mass locations and moments of inertia are expressed in the global coordinate frame. This frame has its origin at the bottom center of solid **s0**, and is aligned with the local frame of segment **P** when somersault, tilt, and twist are zero. In the cases of individual segments or solids, inertial properties are expressed in either the local frame of the particular segment or in the fixed frame. Inertial properties for any other combination are expressed in the fixed frame.

## Usage

We begin our description of how one uses yeadon with an example of an ice skater performing a spin. As is commonly taught in high school physics, an ice skater can change his angular velocity by altering their moment of inertia due to conservation of angular momentum. By what factor can an ice skater increase his angular velocity by bringing in his arms? The commands in Listing 1 perform this calculation.

**Figure 6.**
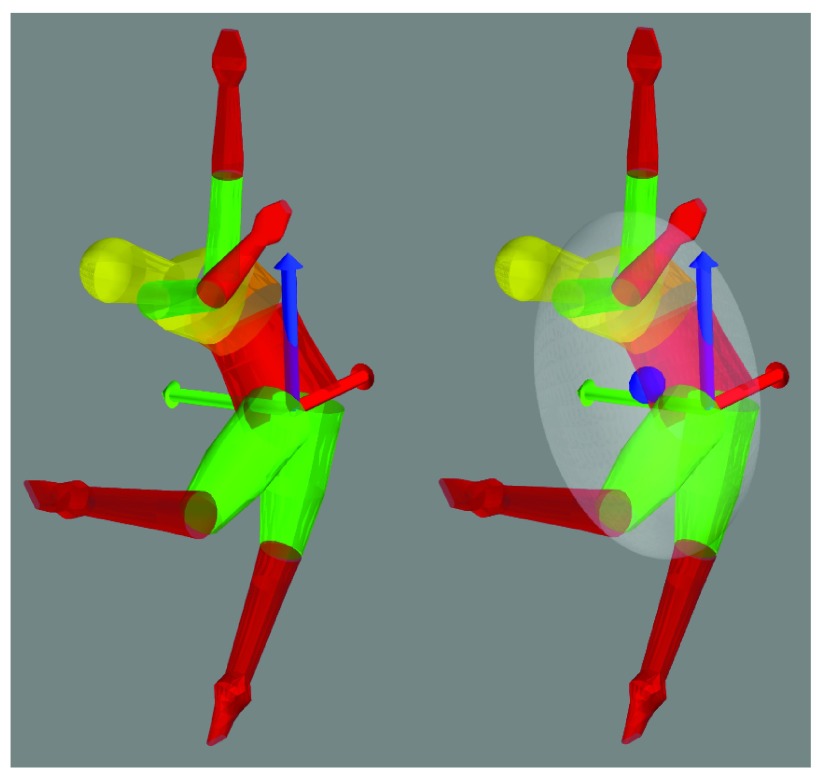
View of the ice skater model in an asymmetrical spin pose. An ice skater can increase the axial moment of inertia by extending their limbs away from the spin axis. The right image shows both the center of mass sphere and the inertia ellipsoid for this pose. Note that the center of mass is actually outside of the body for the gravitational force resultant to be directed through the ground contact point.

The subject represented by the measurements in male1.txt can increase his angular velocity (about the vertical axis) by a factor of 2.9 by bringing the arms in toward the body from an extended position. Figure 6 shows a rendering of a human in a more complicated and asymmetrical ice skating spin pose to demonstrate that the model is capable of complex configurations. We can also obtain the mass and center of mass location of the whole human with the code presented in Listing 2.

With a measurement of the subject’s actual mass, we can scale all the segmental densities so that h.mass is the same as our experimentally measured mass; see Listing 3.

It is also possible to calculate the combined inertia properties of various segments and/or solids. For example, we can obtain the mass, center of mass location, and inertia tensor of the entire right arm via the code in Listing 4.

All of the methods have rich docstrings accessible via the Python help() function. For example, the previous method’s docstring shows the three returned values in Listing 5.


    
                    >>> 
                    import 
                    yeadon
    
                    >>> 
                    pi 
                    = 3.14159
    
                    >>> 
                    # Load a prepared measurements file.
    
                    >>> 
                    h 
                    = yeadon
                    .
                    Human(
                    ’male1.txt’
                    )
    
                    >>> 
                    # Create a 3D rendering of the model.
    
                    >>> 
                    h
                    .
                    draw()
    
                    >>> 
                    # Print the moment inertia about the global Z axis.
    
                    >>> 
                    print
                    (
                    ’Arms down: {:.2f} kg-m^2’
                    .
                    format(h
                    .
                    inertia[
                    2
                    , 
                    2
                    ])
    
                    Arms down: 0.55 kg-m^2
    
                    >>> 
                    # Set the configuration of the shoulder angle.
    
                    >>> 
                    h
                    .
                    set_CFG(
                    ’CA1adduction’
                    , 
                    -0.5 * 
                    pi)
    
                    >>> 
                    h
                    .
                    set_CFG(
                    ’CB1abduction’
                    , 
                    0.5 * 
                    pi)
    
                    >>> 
                    # Print the updated moment inertia about the global Z axis.
    
                    >>> 
                    print
                    (
                    ’Arms out: {:.2f} kg-m^2’
                    .
                    format(h
                    .
                    inertia[
                    2
                    , 
                    2
                    ]))
    
                    Arms out: 1.58 kg-m^2
                


**Listing 1. Python interpreter session showing how one could compute the spin moment of inertia of an ice skater in two configurations.**


    
                    >>> 
                    h
                    .
                    mass
    
                    58.200488588422544
    
                    >>> 
                    h
                    .
                    center_of_mass
    
                    array([[ -9.53791842e-19],
           [  0.00000000e+00],
           [  3.77172308e-02]])
                


**Listing 2. Python interpreter session demonstrating accessing the attributes for mass and center of mass.**


    
                    >>> 
                    # Print the mass of the left leg.
    
                    >>> 
                    h
                    .
                    K1
                    .
                    mass
    
                    8.5047753220376521
    
                    >>> 
                    # Scale the density values based on the measured mass.
    
                    >>> 
                    h
                    .
                    scale_human_by_mass(
                    60.0
                    )
    
                    >>> 
                    # The overall mass is updated.
    
                    >>> 
                    h
                    .
                    mass
    
                    60.0
    
                    >>> 
                    # A slight increase in the segment mass can be seen.
    
                    >>> 
                    h
                    .
                    K1.
                    mass
    
                    8.767736005291324
                


**Listing 3. Python interpreter session which demonstrates segment density scaling.**


    
                    >>> 
                    # Generate the mass, center of mass, and inertia tensor for the right arm.
    
                    >>> 
                    h
                    .
                    combine_inertia([
                    ’B1’
                    , 
                    ’B2’
                    ])
    
                    Combining segments/solids [’B1’, ’B2’].
    (2.8193675676892624,
     array([[-0.1515    ],
            [ 0.        ],
            [ 0.19831659]]),
     matrix([[ 0.09098096,  0.        ,  0.        ],
             [ 0.        ,  0.09110269,  0.        ],
             [ 0.        ,  0.        ,  0.00194811]]))
                


**Listing 4. Python interpreter sessions which demonstrates collecting inertial properties of multiple segments.**


    
                    >>> 
                    help(h
                    .
                    combine_inertia)
    
                    Returns the inertia properties of a combination of solids
    and/or segments of the human, using the fixed human frame (or the
    modified fixed frame as given by the user). Be careful with inputs:
    do not specify a solid that is part of a segment that you have also
    specified. This method does not assign anything to any object
    attributes (it is ’const’), it simply returns the desired quantities.

    See documentation for description of the global frame.

    Parameters
    ----------
    objlist : tuple
        Tuple of strings that identify a solid or segment. The
        strings can be any of the following:

        * solids: ’s0’ through ’s7’, ’a0’ through ’a6’, ’b0’ through ’b6’,
          ’j0’ through ’j8’, ’k0’ through ’k8’
        * segments: ’P’, ’T’, ’C’, ’A1’, ’A2’, ’B1’, ’B2’, ’J1’, ’J2’,
          ’K1’, ’K2’

    Returns
    -------
    combined_mass : float
        Sum of the masses of the input solids and/or segments.
    combined_COM : np.array (3,1)
        Position of the center of mass of the input solids and/or segments,
        expressed in the global frame .
    combined_inertia : np.matrix (3,3)
        Inertia tensor about the combined_COM, expressed in the global frame.
                


**Listing 5. An example docstring for a method in the Human class.**

## Advanced example

This software was originally developed as part of an effort to easily compute the inertial properties of a human rider seated on a bicycle. A common way to model the bicycle/rider dynamics is to assume that the rider is rigid and fixed to the bicycle rear frame ^18^. Our studies ^19^ included a variety of bicycles and riders, for which various combinations of the inertial properties of the bicycle rear frame and rider were required.

As an advanced example, we will configure the model using the yeadon software such that the human is seated on the bicycle, feet at the bottom bracket axis, and hands on the handlebars with arms hanging down. The inertia of the human will be computed first with respect to its center of mass and then combined with that of the bicycle rear frame using the parallel axis theorem to give the total inertia of the human rigidly affixed to the bicycle rear frame in the bicycle’s reference frame.

We use the definitions and parameters of the benchmark bicycle model ^18^ defined using the standard SAE vehicle coordinate system (which is different from Yeadon’s coordinate system). In addition to the geometrical parameters in the benchmark bicycle, the bicycle rear frame and handlebar location are defined by several geometric and inertial parameters. Furthermore, a single measurement of the rider’s forward lean angle relative to the bicycle frame was made.

An interactive IPython ^20^ notebook in the supplementary materials provides a detailed walk through this advanced example. This example is also included in the Yeadon 1.2.1 software source files and a rendered version is viewable with NBViewer. The following briefly summarizes the steps involved in the computation and further illustrates use of the yeadon software.

1. Geometric and inertial properties of the bicycle were estimated with an independent method ^19^. Inertial properties of the rear frame of the bicycle were expressed in the SAE coordinate system described above.
2. We solve for a configuration of the human that enforces the human rider to be seated properly on the bicycle, for any bicycle. The SymPy Mechanics package ^21^ is used for these computations.
3. The Human.inertia_transformed method is used to express the human’s inertia in the bicycle’s reference frame.
4. The combined mass and center of mass location of the human and the rear frame of the bicycle are computed and the parallel axis theorem is employed to express the moments of inertia of each body about the combined center of mass, where they are then summed to get the combined moments of inertia.

The example shows how to set a complex configuration of the Yeadon model and extract the geometric and inertial properties expressed in arbitrary reference frames and relative to arbitrary points as well as how to visualize the configuration.

## Conclusion

We have presented an open source software package that implements a widely used inertial model of a human. This package is available in public repositories under a permissive copyright license. The software provides an API for use as a library, and also has both a command-line user interface and a graphical user interface for interactive high level use as a standalone application. The structural design of the software is presented as an introduction to the source code which is available in a public repository that is open for contributions and modifications. Finally, we have described both simple and advanced use cases for the API, one in the text of the paper and one in the supplementary IPython notebook.

## Software availability

### Software access

http://pypi.python.org/pypi/yeadon/

### Latest source code

https://github.com/chrisdembia/yeadon/tree/v1.2.1

### Source code as at the time of publication

https://github.com/F1000Research/yeadon/releases/tag/V1.2.1

### Archived source code as at the time of publication

http://dx.doi.org/10.5281/zenodo.15770 ^22^

### Software license

yeadon is licensed under the 3 clause BSD license which permits both non-commercial and commercial use.

### Software versions

All of the computations in the paper were executed with the following software versions:

- Python 2.7.9
- IPython 2.3.1
- yeadon 1.2.1
- NumPy 1.9.1
- pyyaml 3.11
- Mayavi 4.3.1
- SciPy 0.15.1
- SymPy 0.7.6
