# Supplementary material for: An object oriented implementation of the Yeadon human inertia model
Source: F1000Res. 2015 Apr 7;3:223. Originally published 2014 Sep 17. [Version 2] doi: 10.12688/f1000research.5292.2 (PMC4329601; doi:10.12688/f1000research.5292.2)
Supplement: Peer review history

# Peer review: Referee response for version 1

**DOI:** 10.5256/f1000research.5643.r6948

**Published:** 2015-02-02

**Authors:** Arnaud Barré

**Affiliations:** 1 Département de génie de la production automatisée, Ecole de Technologie Superieure, Montréal, QC, Canada

**General comments**

The aim of this manuscript is to present a software tool used to:

 1. facilitate the computation of the body segment inertial parameters proposed by M.R. Yeadon;

 2. visualize in three dimensions the posture of an avatar;

 3. request/compute some body segment inertial parameters related to the posture set.

The description of the Yeadon's model is very well detailed as well as all the modifications realized. The illustrations and examples presented shows also the possibilities of the software.

However, it would be interesting to have a better comparison of the different methods proposed to compute body segment inertial parameters using direct and indirect methods. For example, the authors mentions the facility of Yeadon's method compared to other studies, but the number of measurements is large compared to others studies not cited, like de Leva (1996) or Dumas *et al.* (2007). These indirect methods have also error as explained in Rossi *et al.* (2013). What is the advantage of Yeadon's method compared to others? A discussion on the limitations of the model proposed (joint center accuracy, joint range, mass estimation, density scaling, etc.) would be also of interest. The authors modified some parts of the original model, why did they not add joint centers for ankles and wrists? This would give a better general behavior to the avatar.

**Specific comments**

I would suggest to the authors to clarify the points listed below:

Figure 1: The authors should give the abbreviations of the body parts. It is not obvious why all the levels for the torso does not use the P,T,C letters, but 's' instead. I understand this is the original nomenclature used by M.R. Yeadon, but this should be detailed. Moreover, as explained later in the manuscript, some segments have two levels at the same location (for example, shoulder and neck levels are abbreviated by Ls5), they would be distinguished for clarity. Thus, this would help to better understand the departures proposed (like in Acromion stadia).

Figure 1: In the legend "segments, label, solids", the segment A2 is defined between a2 and s6. I assume it is a6 and not s6.

"Measurements": fifth paragraph: The authors should mention the potential accuracy issue related to the scaling of the densities (homogeneous scaling over the body, possibly increasing the error with the body segment inertial parameters). This could be in the discussion.

"Configuration" paragraph 1: The authors wrote "somersalt" instead of "somersault". (Also in page 7).

Formulas 1 and 2: These formulas give only the x coordinates of the hip center joint. What about the y and z coordinates?

Formulas 3, 4, 5: The authors should use a suffix to the computed parameters to clarify the associated level. 

"Relationships between configuration variables": The authors wrote "21 configuration variables;". Please modify to "21 joint angle configuration variables to clarify".

"Degenerate Stadia": What are the manipulations? Please detail.

"Software design": Please add a reference for "Python" as this is the only one tool that does not have one (compared to Mayavi or VTK). The authors should also indicate that the library is implemented with Python 2.7. Their code could not be compatible with Python 3.

Figure 5: How did the authors fix the joint angular range? This seems to be empirical but it should be at least mentioned in the manuscript.

I have read this submission. I believe that I have an appropriate level of expertise to confirm that it is of an acceptable scientific standard.

# Peer review: Referee response for version 1

**DOI:** 10.5256/f1000research.5643.r6185

**Published:** 2014-10-30

**Authors:** Alison L. Sheets

**Affiliations:** 1 Nike Sports Research Lab, Beaverton, OR, USA

The aim of this manuscript is to describe a software tool that was developed to calculate the inertia properties of the human body and segments of the body using the method developed by M. R. Yeadon. Yeadon's method represents the body as a series of solid shapes, and is frequently used in biomechanical analyses. The software described in this paper makes the method developed by Yeadon faster and easier to implement.  

The manuscript is very well written, includes numerous examples that highlight the software flexibility and capabilities, and the host site for the software is well documented. Additionally, the detailed description of the inertia model clarifies a few ambiguities from Yeadon’s original text, and modifications were made to enable the model to answer more general questions. The figures are detailed and descriptive.

My only minor suggestion is for the authors to add definitions for terms in Figure 4 that are not self-descriptive. For example, it is not clear what rel_center_of_mass and rel_inertia are defined relative to.

I have read this submission. I believe that I have an appropriate level of expertise to confirm that it is of an acceptable scientific standard.

# Response

**Published:** 2014-11-05

**Authors:** Jason Moore

**Affiliations:** Cleveland State University, USA

Thank you for the review and the approval. As far as the method/attribute names are concerned in the UML diagram (Fig 4), we specifically didn't include great detail, trying to focus on the object hierarchy and the the most important methods/attributes of the objects. All of the methods and attributes are explained in the online software documentation, for example:

http://yeadon.readthedocs.org/en/latest/segment.html

We can add some of those details in the paper if you think it will clear up things, or maybe link to the online documentation. I will discuss with the co-authors.
